# Resistance to kinase inhibition through shortened target engagement

**DOI:** 10.1080/23723556.2022.2029999

**Published:** 2022-01-22

**Authors:** Aziz M. Rangwala, Benedict-Tilman Berger, Matthew B. Robers, Stefan Knapp, Markus A. Seeliger

**Affiliations:** a Department of Pharmacological Sciences, Stony Brook University Renaissance School of Medicine, Stony Brook, NY, USA; b Institute of Pharmaceutical Chemistry, Goethe University Frankfurt, Frankfurt am Main, Germany; c Structural Genomics Consortium, Buchmann Institute for Life Sciences, Goethe University Frankfurt, Frankfurt am Main, Germany; d Research and Development Department, Promega Corporation, Fitchburg, WI, USA

## Abstract

Imatinib, a selective inhibitor of the breakpoint cluster region (BCR)-ABL kinase, is the poster child for targeted cancer therapeutics. However, its efficacy is limited by resistance mutations. Using a quantitative bioluminescence resonance energy transfer assay in living cells, we identified ABL kinase mutations that could cause imatinib resistance by altering drug residence time.

Protein phosphorylation, catalyzed by protein kinases, is a critical signaling process essential to life. Dysregulation of this post-translational modification drives the pathogenesis of many human diseases including autoimmune, cardiovascular, nervous system disorders, and cancer. It was originally thought that targeting members of the kinome with adenosine triphosphate (ATP)-competitive small molecules was infeasible due to high sequence conservation of the ATP-binding site. The discovery of imatinib, an inhibitor of the breakpoint cluster region (BCR)-ABL oncoprotein, upended this myth. BCR-ABL, a fusion between the breakpoint cluster region and ABL kinase, is the major driver of leukemogenesis in chronic myelogenous leukemia (CML). Imatinib reduced the mortality rate of CML by 80% within a decade of its approval. Consequently, imatinib became the poster child for specific kinase inhibition and spawned an entire field of kinase-targeted drug discovery. Roughly a third of modern drug discovery efforts are focused on small-molecule modulators of kinases.

Active kinases adopt strikingly similar poses when catalyzing phosphotransfer with their substrates. However, kinases are not just static phosphorylation machines; they are dynamic proteins that interconvert between a large ensemble of inactive conformations and a more conserved active conformation.^1,2^ Imatinib achieves its high specificity in part by binding preferentially to an inactive conformation of ABL kinase, whereas the more promiscuous inhibitor dasatinib binds to the active conformation. Furthermore, conformational dynamics affect both the drug-binding process^3^ and dissociation process,^4^ thereby providing a vulnerability for therapeutic intervention and small-molecule design.

Drug action in the body is tied to dynamic processes such as absorption, distribution, metabolism, and excretion (ADME). Therefore, dissection of the equilibrium binding constant *K*_d_, the standard measure of a lead compound’s efficacy, into the rate constants for drug binding to the target (*k*_on_) and dissociation from the target (*k*_off_) may be more relevant for non-equilibrium *in vivo* systems. Residence time, the reciprocal of the drug dissociation rate constant, describes the lifetime of the drug-target complex and has been shown to be a superior predictor of *in vivo* potency in several systems.^5^ Drug dissociation rates have been of particular interest to kinase inhibitor development since the discovery of BIRB796, a reversible p38α mitogen-activated protein kinase inhibitor with an unusually long estimated residence time of >1800 hours.^6^ The analysis of 270 kinase inhibitors against 40 kinases revealed an increasing number of slow-dissociating compounds when transitioning from early/preclinical compounds to late stage and FDA-approved compounds.^7^ Kinetic selectivity, where the drug-target complex has a longer half-life than off-target-drug complexes, is an emergent strategy to achieve specific kinase inhibition. For example, we modified a series of aminopyrimidines to create inhibitors with much longer residence times for focal adhesion kinase over the closely related proline-rich tyrosine kinase 2.^8^

This led us to ponder: if chemical modifications to inhibitors can improve their efficacy by prolonging residence time, could the reverse be true for mutations in kinases that reduce inhibitor efficacy by reducing residence time?

In our recent study, Lyczek et al.,^9^ we used NanoBRET,^10^ a bioluminescence resonance energy-transfer (BRET) technique that quantitatively assesses target engagement in live cells, to test the affinity and dissociation kinetics of imatinib and dasatinib against a library of 94 ABL kinase domain mutations. These mutations were observed in CML cases with resistance to imatinib therapy. Surprisingly, we found that two-thirds of mutations significantly reduce drug affinity while one-third of mutations retain similar or tighter affinity to wild-type (WT). This posed the question: how could 31 mutations that have similar binding affinity to imatinib cause imatinib resistance? We found mutations at three sites, N368S, V299L, and G251E with significantly faster imatinib dissociation rates. These sites are frequently altered in other kinases and cancer types: there are nine mutations across eight kinases reported for N368, three mutations across three kinases reported for V299, and 13 mutations across eight kinases reported for G251. We further validated the observed effect of the N368S mutation *in vitro* and proposed a mechanism of its increased dissociation rate using molecular dynamics simulations.

In contrast to thermodynamic mutations that decrease the imatinib binding affinity, kinetic mutants increase the drug off-rate and on-rate while maintaining a similar affinity to WT (Figure 1(a)). While thermodynamic mutations never reach a threshold for pharmacological effects, kinetic mutants instead decrease the lifetime of the imatinib-ABL kinase complex, potentially reducing drug efficacy between doses (Figure 1(b)). Patients with kinetic mutations are still sensitive to drug therapy and could potentially respond by modifying the treatment schedule from a single daily dose to multiple doses.

This study represents the first comprehensive comparison of the effect of disease-relevant mutations on drug affinity and binding durability in live cells. Mutations at these sites are present ubiquitously throughout the kinome, which suggests that kinetic resistance to structurally selective kinase inhibitors may be a widespread mechanism. We envision that similar investigations of mutations in other drug-target pairs will uncover a broader role for binding kinetics in drug resistance and impact clinical standards of care.

**Figure 1.**
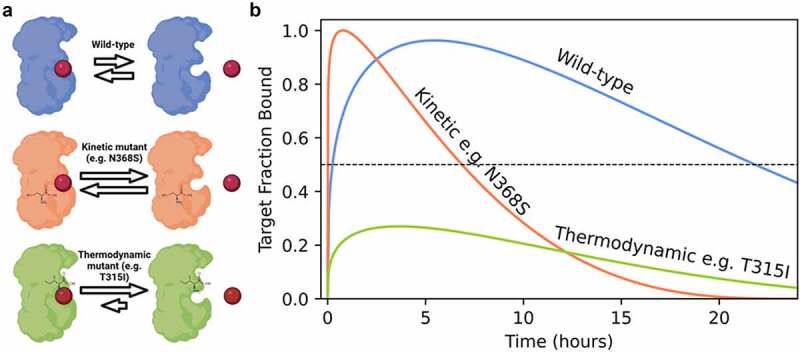
Altering drug binding rates may be a partial resistance mechanism to kinase inhibition. (a) Kinetic mutations in breakpoint cluster region (BCR)-ABL cause resistance through increased drug binding and dissociation rates, whereas thermodynamic mutations abrogate drug binding. (b) Simulated effect of mutation on compound off-rates in a model system of a patient over 24 h. Threshold for pharmacological inhibition is 50% of target fraction bound. Coloring scheme consistent with panel A.

**Alt Text**: (a) Three cartoon breakpoint cluster region (BCR)-ABL proteins binding to a spherical cartoon drug with differently sized rate arrows to illustrate wild-type versus a kinetic mutant and a thermodynamic mutant. (b) Simulated effect of mutation on residence time. Three curves plotted with a dotted line representing the fraction of target protein bound necessary for inhibition. Wild-type stays above the dotted line for the full duration, whereas the kinetic mutant stays above the dotted line for only a third of the duration and the thermodynamic mutant never reaches above the line.
